# Nitrate of Silver in the Treatment of Uterine Polypi

**Published:** 1868-11

**Authors:** M. M. Eaton

**Affiliations:** Peoria, Ill., Membre Adhérent du Congress Médical International de Paris


ARTICLE XLV.

### NITRATE OF SILVER IN THE TREATMENT OF UTERINE POLYPI.

By M. M. EATON, M.D., Peoria, Ill., Membre Adhérent du Congress Médical International de Paris.

Mrs. D., aged 46 years, living 25 miles in the country, consulted me in June, 1866, regarding a uterine hemorrhage, which had annoyed her for over two years, almost constantly.

Upon examination, I found her of a nervous temperament, anæmic, emaciated. By digital examination, I found two polypi, of the size of a filbert, external to the uterus, and attached by a long pedicle to the margin of the os uteri. I explained the case to my patient, and recommended their removal by torsion. She consented, but desired that I should come to her home to remove them, and promised to write in about two weeks what day I should come. I gave her a tonic of bark and iron, and heard no more of the case for about a year, when she again consulted me, June 25th, 1867. She stated, that soon after her visit to me last year, she had discharged the two small tumors per vaginam, and that she had been much improved in general health, and that the hemorrhage had ceased for two or three months, and she had not sent for me for these reasons; but that now she was troubled with the hemorrhage as badly as ever, and had been for eight or nine months; and, as she was getting worse, she desired something should be done. I requested her husband to place her in a boarding-house, and let her remain a while in the city, which he did. She was much exhausted with her ride in the car, and I delayed a vaginal examination till the next day.

*June 26th.*—On making a digital examination, I could detect no tumors, and nothing abnormal, except an enlargement of the womb, and considerable hemorrhage, mixed with pus of an unhealthy character. On attempting to examine the uterus with a uterine sound, there was such an alarming hemorrhage as to cause me to desist before ascertaining anything, except that the canal seemed to be obstructed. I immediately introduced a sponge-tent, to serve as a tampon and dilate the neck of the uterus (which, I should have stated, was nearly closed). This arrested the hemorrhage, but caused much pain. I gave anodynes, and introduced a larger tent, and so on, for four days, when, on passing my finger into the neck of the womb, I detected the whole surface thickly studded with polypi, from the size of a grain of wheat to that of a kernel of corn, and three or four about the size of a hickory-nut, situated high up.

The hemorrhage was free from the examination, and I concluded I would apply the solid nitrate of silver, and again introduce the sponge-tent, which acted well as a tampon. I accordingly did so, cauterizing all the tumors freely, through a duck-billed speculum, endeavoring to touch the pedicles of all I could.

The next day removed the sponge, and several small polypi (glandular variety) were discharged. I again applied the silver, and left the womb open. On the fourth day of the use of the caustic the hemorrhage had almost ceased, and I found that the tumors in the neck had all been detached and discharged, but there was considerable discharge of offensive matter. I syringed the uterus with tepid water, in which there was a small amount of liq. chlo. sodæ, and applied the silver to those suppurating spots where the pedicles had been attached, which, by the way, constituted nearly the whole internal surface of the neck; keeping her under the influence of anodynes most of the time, and giving tonics, with beef-tea and soup.

Continued this treatment ten days, when she commenced to flow immoderately, and I made another digital examination, and found two tumors in the cavity of the womb, apparently about the size of an orange. I now gave a decoction of ergot, which caused uterine contractions and some slight descent of the tumors. My patient's strength giving way, I feared to delay matters, and applied caustic to the tumors as freely as possible. This, with the ergot, arrested most of the hemorrhage, and I contented myself with giving tonics, anodynes, and nourishment, and applying the silver every two or three days, for nearly two weeks, when, considerable pain coming on, I made an examination, and found I could brake them down with my finger; which I did, using forceps two or three times, to assist, when I was enabled to get the tumors entirely away, leaving the uterus entirely empty, but considerably enlarged.

I again cauterized the seat of the attachment of the pedicles, which were about ½ inch in diameter, and gave decoction of ergot. The uterus contracted kindly, and patient expressed herself as feeling greatly relieved. August 10th, three days after, found the uterus contracted, but considerable discharge of tolerably healthy pus. Gave the phos. elix. bark and iron, in ʒ doses, four times a day, and recommended bathing with tepid water and open-air exercise in a carriage for a short distance each day.

*September 3d.*—Case has progressed favorably: no hemorrhage or matter for several days; health improving; appetite good. Allowed her to return home.

*July 9th, 1868.*—Saw Mrs. D. Is now doing her own work; has had no hemorrhage; matter ceased, excepting a leucorrhœal discharge sometimes. Made an examination, to satisfy myself of the condition of affairs, and found everything normal except a slight prolapse of the uterus. Recommended the wearing of an abdominal supporter, which will probably rectify the slight misplacement. So far as the tumors are concerned, she must be considered cured; and she says her general health is as good as she could expect, as she has always been weakly. She has gained very much in flesh since last year, and has quite good color.

I made a note of this case from the comparative simplicity of the treatment, it not requiring many instruments or much skill, hoping that physicians in small places will take hold of these cases resolutely, instead of giving a palliating and a dubious prognosis, many times making no examination of the uterus, but simply taking a patient's word for the state of affairs. I might occupy pages in telling amusing anecdotes of the mistakes ladies have made respecting their own cases, which have occurred in my limited experience, but this is foreign to my subject.

My principal reason for employing the nitrate of silver was to prevent hemorrhage, which I feared would prove fatal if I used torsion, as I have usually done in small polypi (I have successfully operated on two cases the last month, using torsion for removing a single polypus in each case), and also the favorable result in a case I reported on page 1 of this volume, in the destruction of a large watery tumor or hydatid, which had caused retroversion and inability to use one leg for two years.

Besides, Dr. Montgomery, in his paper published of "Uterine Tumors," says "small polypi may be conveniently destroyed by caustic."

Prof. Byford also states, on page 370 of his work on "Diseases of Women," that "Dr. Simpson, of Edinburgh, has at least in one instance effected the destruction of a uterine tumor by cauterizing its interior with potassa fusa."

I simply give this little experience with "argentum nitratis," thinking it to be more convenient and less dangerous than potassa fusa, and perhaps, in cases like the one just named, more applicable than either torsion or excision; or removal by ligature or the écrasseur; or even by the new method of Prof. Byford, of Chicago, "by introducing a dossil of cotton or lint through a canula into the substance of the tumor; the cotton to remain and produce suppurative action, and be removed by means of a thread tied to it before its introduction." His method, however, may be useful in some cases, especially where the tumor is solitary.

---

THE PHILADELPHIA MEDICAL REGISTER AND DIRECTORY.—We take pleasure in announcing the appearance of this useful little volume, edited by Dr. John H. Packard, and which has been recently issued by Collins, Printer, 705 Jayne Street. It furnishes much useful information regarding the various Medical Associations, Medical Schools, and kindred Institutions; Hospitals, Dispensaries, Charitable Institutions, and other matters, and a Directory of the Physicians of Philadelphia, with their office hours.—*Medical News and Library*, October, 1868.

